# MircoRNA-145 promotes activation of hepatic stellate cells via targeting krüppel-like factor 4

**DOI:** 10.1038/srep40468

**Published:** 2017-01-16

**Authors:** Ruoting Men, Maoyao Wen, Mingyue Zhao, Xuelian Dan, Zongze Yang, Wenchao Wu, Maggie Haitian Wang, Xiaojing Liu, Li Yang

**Affiliations:** 1 Division of Gastroenterology & Hepatology, West China Hospital, Sichuan University, Chengdu 610041, China; 2 Department of Biostatistics, JC school of Public Health and Primary Care, Faculty of Medicine, The Chinese University of Hong Kong, China; 3 Laboratory of Cardiovascular Diseases, Regenerative Medicine Research Center, West China Hospital, Sichuan University, Chengdu 610041, China; 4 Creation and Management of a Tumour Bank, West China Hospital of Sichuan University, Chengdu, Sichuan 610041, China

## Abstract

Krüppel-like Factor 4 (KLF4), a target gene of miR-145, can negatively regulate lung fibrosis. However, the potential role of KLF4 and miR-145 in hepatic stellate cells (HSCs) activation or in hepatic fibrosis keeps unclear. This study aims to characterize miR-145 and KLF4 in activated HSCs and liver cirrhotic, and the underlying molecular basis. miR-145 was significantly up-regulated, while KLF4 was dramatically down-regulated during the activation of rat primary HSCs and TGF-βtreated HSCs. Furthermore, miR-145 mimics induced and inhibition of miR-145 reduced α-SMA and COL-I expression in primary HSCs. Additionally, the mRNA and protein levels of KLF4 in the liver of cirrhotic patients and rats were significantly down-regulated. α-SMA and COL-I were increased after inhibition of KLF4 by specific shRNA in primary HSCs. Forced KLF4 expression led to a reduction of α-SMA and COL-I expression in HSCs. miR-145 promotes HSC activation and liver fibrosis by targeting KLF4.

Hepatic stellate cells (HSCs) play a key role in liver fibrosis/cirrhosis. Activated HSCs are the major cell type responsible for excessive deposition of extracellular matrix (ECM) components. Upon liver injury, HSCs transdifferentiate into myofibrolast-like cells marked with α-smooth muscle actin (α-SMA) expression, ECM production, increased proliferation and contractive ability^1^,^2^. However, the mechanism of HSCs activation remains largely unknown.

Recently, miRNAs have emerged as pivotal regulators in the activation of HSCs^3^,^4^. MicroRNAs (miRNAs) are small non-coding RNAs with a negative role in target gene translation. MiR-122 regulates activation of HSCs and liver fibrosis by controlling collagen maturation and ECM production^5^. Ogawa *et al*. reported that miR-29b directly regulated collagen synthesis via binding to the 3′UTR of collagen and transcriptional regulator SP1 in a human HSC cell line^6^. MiR-143/145, encoded as a gene cluster^7^, play a crucial role in fibrotic diseases. MiR-145 is elevated in cystic fibrosis and lung fibrosis^8^,^9^. Elevated miR-145 increased α-SMA by target KLF4 in lung fibroblasts^8^. MiR-143 enhanced collagen-III expression in stromal fibroblasts of scirrhous type gastric cancer^10^ and is increased in activated HSCs^11^. The role of miR-145 in activation of HSCs is still to be demonstrated.

Krüppel-like factor (KLF) 4, also known as gut-enriched KLF/GKLF or epithelial/endothelial zinc factor/EZF, belongs to a sub-family of zinc-finger class of transcriptional regulators^12^,^13^. Other important member of KLF family, KLF2 has been reported to be an essential regulator in the activation of HSCs^14^. Moreover, there was a compensatory increase in KLF4 expression upon KLF2 knockdown which suggested a potential role of KLF4 in pathogenesis of liver fibrosis. Villarreal *et al*. reported that KLF2 and KLF4 have a functional similarity by regulating same target genes^15^. In vascular smooth muscle cells, KLF4 repressed α-SMA expression by bound to the TGF-β control element (TCE) at the promoter of α-SMA^16^. However, other reports showed a promotional role in α-SMA expression^17^. To the best of our knowledge, the role of KLF4 in HSCs activation has not been reported. Given the effect of KLF4 on α-SMA transcription, the role of KLF4 in activation of HSCs and in hepatic fibrosis/cirrhosis remains to be determined.

In the present study, we measured the character of miR-145 in HSCs activation and its regulatory role in KLF4 expression. Moreover, we determined the role of KLF4 in HSCs activation and liver fibrosis.

## Results

### Elevated expression of miR-145 in spontaneously and TGF-β induced activation of HSCs

Our previous study has reported a total 53 miRNAs between quiescent and activated HSCs by microRNA microarray analysis^18^. There are some miRNAs that have been reported to be differentially down-regulated (e.g., miR -29c, -195, -652) or upregulated (miR-146a, -21, miR-143, etc) in activated HSCs, indicating the expression level of miRNA were consistent at certain degree in the process of HSC activation. MiR-145 is one of the upregulated miRNA among the reported 53 miRNAs.

**Figure 1:**
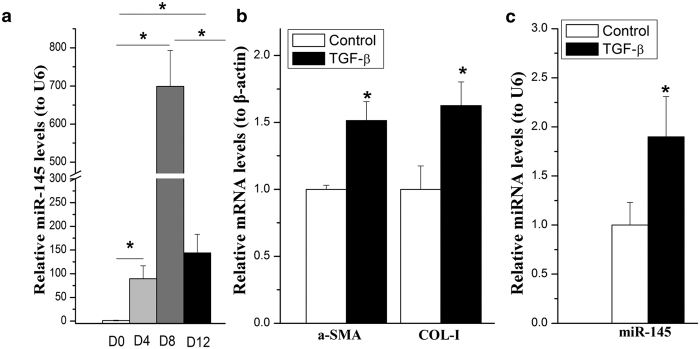
miR-145 was up-regulated in spontaneously and TGF-β activated HSCs. The miRNA level of miR-145 or mRNA levels of α-SMA or COL-I were examined by qRT-PCR. (**a**) miR-145 was examined by quantitative RT-PCR (qPCR) in the process of HSCs’ spontaneously activation. (**b**) The mRNA levels of α-SMA and TGF-β1 in HSCs treated with TGF-β. (**c**) Induction of miR-145 expression in rat primary HSCs by TGF-β stimulation. All experiments were repeated thrice with triplicate samples in each experiment. The relative value of miR-145 to U6 (or target mRNA to β-actin mRNA) was set as 1 in the control or day 0 HSCs. Data are presented by mean ± standard deviation. *p < 0.05.

To assess the function of miR-145 in activation of HSCs, we detected the expression of miR-145 in quiescent (day 0), semi-activated (day 4) and completely-activated (day 12 and passaged) HSCs by qRT-PCR. We confirmed that miR-145 was indeed increased during the process of HSCs activation. MiR-145 showed an obviously increase near 100-fold in day 4 and 700-fold in day 12, respectively (Fig. 1a). It was interesting that the expression level miR145 was significantly decreased after passages as compared to day 12, although it still kept at high level (Fig. 1a).

TGF-β is one of the most potent pro-fibrogenic mediators in HSC activation as evidenced by stimulation of collagen production^19^,^20^,^21^. To determine whether TGF-β modulates miR-145 expression, the activated rat primary HSCs (within 3–5 passages) were further activated by treating with 10 ng/ml of TGF-β1. As shown in Fig. 1b and c, in line with the increased α-smooth muscle actin (α-SMA) and COL-I levels (Fig. 1b), miR-145 (Fig. 1c) was up-regulated by TGF-β1 stimulation.

Together, our data showed that miR-145 were significantly up-regulated both in the process of culture activation of primary HSCs and in TGF-β induced HSC activation, suggesting an important role of miR-145 in liver fibrosis.

### MiR-145 level is up-regulated in murine and human liver fibrosis

**Figure 2:**
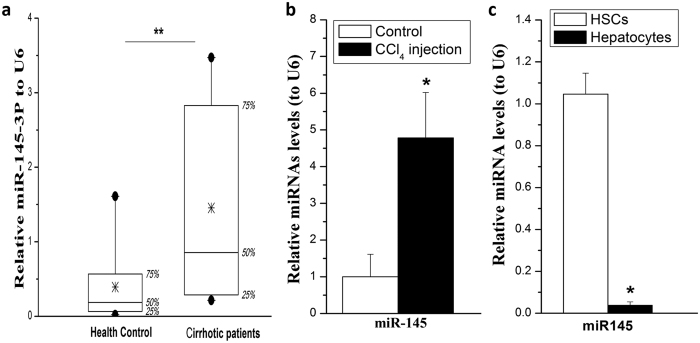
MiR-145 level in liver tissues from hepatic cirrhotic patients and rats. (**a**) miR-145 was significantly increased in cirrhotic liver of patients. (**b**) miR-145 expression was increased in fibrotic liver of rats (n = 6). (**c**) miR-145 levels in normal rat primary hepatocytes and HSCs. All experiments were repeated thrice with triplicate samples in each experiment. The relative value of miR-145 to U6 (or target mRNA to β-actin mRNA) was set as 1 in the control or day 0 HSCs. Data are presented by mean ± standard deviation. *p < 0.05.

To determine if miR-145 were also elevated in fibrotic liver, total RNA was isolated from human cirrhotic and non-fibrotic liver tissues and rat fibrosis models. We firstly detected the miRNA-145 levels in liver tissues from hepatic fibrosis patients (Metavir score = 4). MiR-145 level significantly increased in cirrhotic liver of patients as compared to non-fibrotic controls (Fig. 2a). We also detected miR-145 levels in rat fibrotic livers, and found that miR-145 expression was increased (Fig. 2b).

Both HSCs and hepatocytes are very important in liver fibrosis. To reveal the expression level of miR-145 in hepatocytes and HSCs in the liver, we isolated and purified primary hepatocytes and HSCs from normal rats, and measured miR-145 level. We showed that miR-145 is highly expressed in HSCs rather than in hepatocytes (Fig. 2c).

### Promotion of HSC activation by miR-145

**Figure 3:**
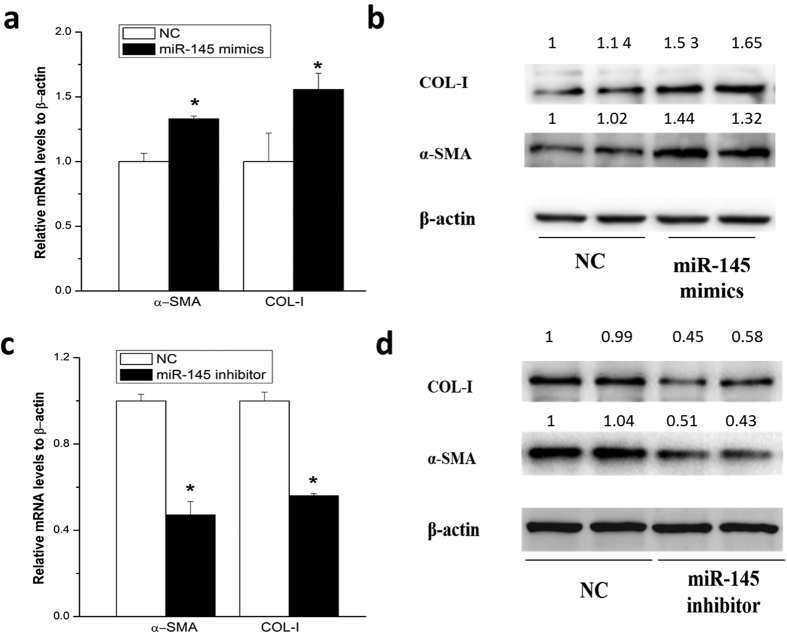
miR-145 promoted HSC activation. The miRNA level of miR-145 or mRNA levels of α-SMA or COL-I were examined by qRT-PCR after transfecion of specific mimics or inhibitors for 48 hours in activated rat primary HSCs. (**a**) The mRNA level ofα-SMA or COL-I after transfecion of miR-145 mimics. (**b**) The protein level ofα-SMA or COL-I after transfecion of miR-145 mimics. (**c**) The mRNA level of α-SMA and COL- I after transfection of miR-145 inhibitors. (**d**) The protein level of α-SMA and COL- I after transfection of miR-145 inhibitors. All experiments were repeated thrice with triplicate samples in each experiment. The relative value of target mRNA/protein to β-actin was set as 1 in the control. Data are presented by mean ± standard deviation. *p < 0.05; **p < 0.01.

To examine whether miR-145 could be able to regulate activation of HSCs, rat primary HSCs were transfected with miR-145 specific mimics or a scrambled miRNA for 48 hours, respectively. The mRNA and protein levels of α-SMA and COL-I, the markers of HSCs activation, were significantly elevated as compared with the control (Fig. 3a).

Furthermore, endogenous miR-145 was down-regulated by transfecting miR-145 specific inhibitor for 48 hours in activated primary HSCs. As a consequence, the mRNA and protein levels of α-SMA and COL-I were significantly down-regulated (Fig. 3c and d).

These results revealed that miR-145 promoted HSC activation.

### Down-regulation of KLF4 is in activated HSCs and cirrhotic liver tissues

**Figure 4:**
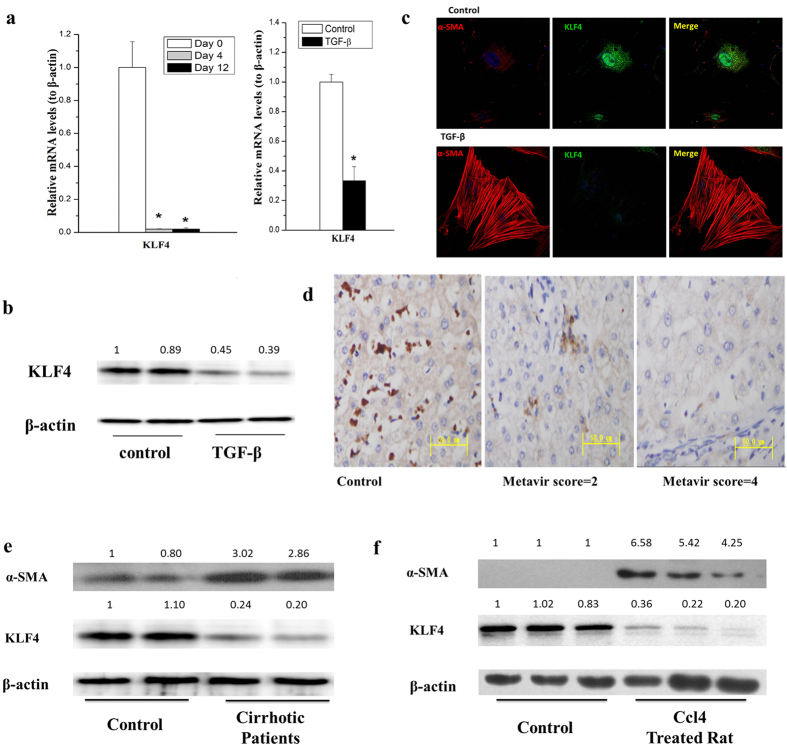
KLF4 was down-regulated in activated HSCs and cirrhotic liver tissues. (**a**) The mRNA level of KLF4 dramaticlly decreased in the process of spontaneous HSC activation (left pannel) and primary HSCs treated with 10 ng/mL of TGF-βfor 24 hours (right pannel). (**b**) KLF4 protein level was significantly decreased in HSCs by treating primary HSC with 10 ng/mL TGF-β for 24 hours. (**c**) TGF-β down-regulated KLF4 while upregulatedα-SMA in rat primary HSCs. KLF4 and α-SMA were detected by immunocytochemical staining and pictures were taken with a confocal microscopy. Rat primary HSCs were treated with 10 ng/mL TGF-β for 4 hours. (**d**) KLF4 was significantly suppressed in cirrhotic liver of patients as compared with the healthy controls (scored by immunohistochemistry studies, n = 16). (**e**) KLF4 was significantly down-regulated, whileα-SMA was obviously up-regulated in human cirrhotic liver tissues compared with the healthy controls. (**f**) KLF4 was elevated in CCl_4_ induced cirrhotic liver of rats. All experiments were repeated thrice with triplicate samples in each experiment. The relative value of target mRNA/protein to β-actin was set as 1 in the control. Data are presented by mean ± standard deviation. *p < 0.05; **p < 0.01.

The status and functions of KLF4 have not yet been investigated in HSC activation and liver cirrhosis. We first checked the mRNA levels of KLF4 in rat primary HSCs by qRT-PCR. We showed that the mRNA level of KLF4 was dramatically declined in both semi-activated and fully activated HSCs as compared with quiescent HSCs (Fig. 4a, left pannel) and TGF-β treated HSCs (passed rat primary HSCs) (Fig. 4a, right pannel), indicating that there were a close association between KLF4 level and HSC activation. To further confirm TGF-β could suppress KLF4 expression, we preformed western blot experiment to check KLF4 proteins. As shown in Fig. 4c, the protein level of KLF4 was obviously decreased by treating with 10ng/mL of TGF-β for 24 hours. In addition, we directly viewed KLF4 protein level and co-localization of KLF4 and α-SMA in TGF-β stimulated rat primary HSCs by immunofluorence staining under a confocal microscope. We showed that KLF4 localized in both nucleus and cytoplasm of HSCs. Obviously, KLF4 was largely suppressed after TGF-β treatment (Fig. 4c).

To exam KLF4 level in cirrhotic livers, immunohistochemistry studies were employed to observe KLF4 protein levels liver tissues from cirrhotic patients (Fig. 4d). Furthermore, proteins from cirrhotic liver specimens of patients were isolated and applied for western blot assays. Compared to non-fibrotic livers, KLF4 significantly decreased in the cirrhotic livers. Obviously, fibrotic marker α-SMA significantly increased in cirrhotic liver specimens (Fig. 4e). Finally, we showed that the protein level of KLF4 significantly decreased in the cirrhotic liver of CCl4 induced rat models (Fig. 4f), similar to that of cirrhotic liver in patients.

### Promotion of HSCs activation by KLF4 knockdown

**Figure 5:**
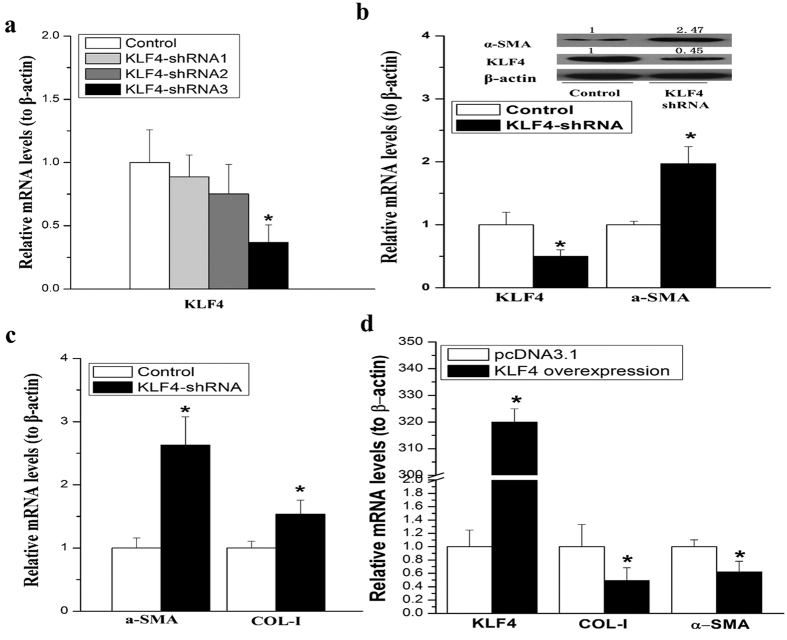
Knockdown of KLF4 promoted TGF-β induced HSC activation. (**a**) KLF4-shRNA decreased the mRNA level of KLF4 in rat HSCs. (**b**) The mRNA levels of α-SMA and COL-I expression were upregulated by knockdown of KLF4. (**c**) KLF4 knockdown by shRNA stimulated α-SMA protein expression. (**d**) Overexpression of KLF4 suppressed transcription ofα-SMA, COL-I and TGF-βmeasused by qRT-PCR. Data are presented by mean ± standard deviation. *p < 0.05; **p < 0.01.

To explore the functions of KLF4 in HSCs activation, we constructed shRNA expressing plasmids (pKLF4-shRNA) to specifically knock down KLF4 in rat HSCs. We designed three pairs of shRNA sequences and a scrambled sequence. Among these three shRNA, the third one was the most effective (KLF4-shRNA) as shown in Fig. 5a. Surprisingly, the mRNA level of α-SMA and COL-I levels were significantly increased in the activated HSCs after silencing KLF4 (Fig. 5b). Consistent to mRNA elevation, α-SMA protein level was also increased by KLF4 knockdown in HSCs (Fig. 5c). On the other hand, overexpression of KLF4 reduced the mRNA levels of α-SMA, COL-I and TGF-β in primary HSCs (Fig. 5d).

### KLF4 is the direct target of miR-145 in HSCs

**Figure 6:**
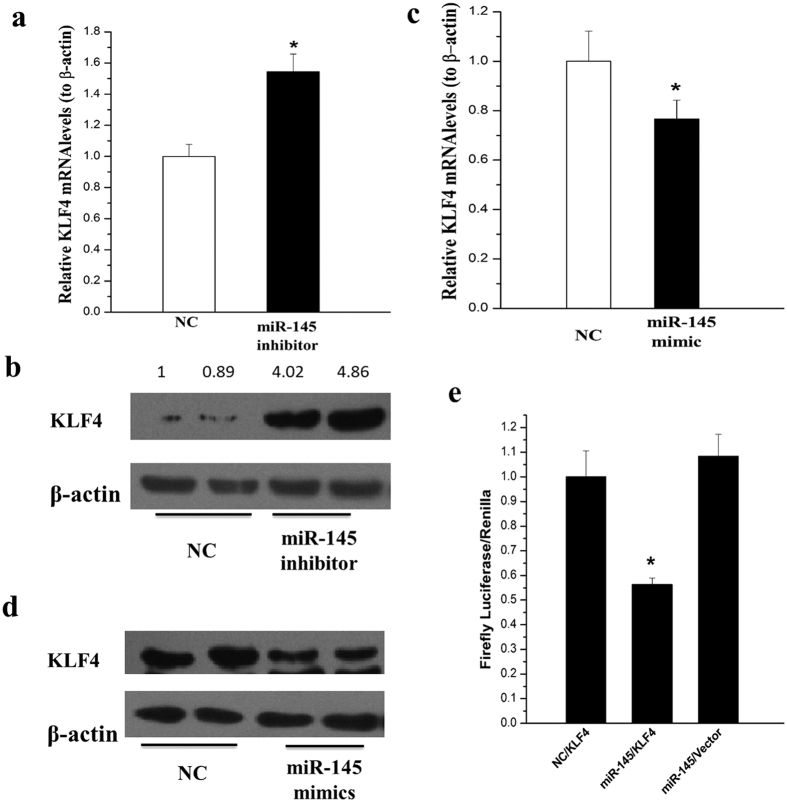
KLF4 was suppressed by miR-145 in HSCs. (**a**) The mRNA level of KLF4 was elevated by miRNA-145 inhibitors. (**b**) The protein level of KLF4 was up-regulated by miRNA-145 inhibitors. (**c**) miRNA-145 mimics descreased KLF4 mRNA level. (**d**) miRNA-145 mimics reduced KLF4 protein level. (**e**) LX2 cells were co-transfected with negative control with 3′UTR of KLF, miR-145 mimics with 3′UTR of KLF4 or miR-145 with pmirGLO Vector respectively. The dual luciferase activity were detected after 48 hours. All experiments were repeated thrice with triplicate samples in each experiment. The relative value of target mRNA/protein to β-actin was set as 1 in the control. Data are presented by mean ± standard deviation. *p < 0.05; **p < 0.01.

Although miR-145 has been reported to target KLF4 in vascular smooth muscle cells and other mesenchymal cells^22^,^23^, its effect on KLF4 have not been examined in HSCs. In this study, we showed that both mRNA and protein levels of KLF4 were increased after transfection of miR-145 inhibitor (Fig. 6a and b); whereas transfection of miR-145 mimics reduced both mRNA and protein levels of KLF4 in rat HSCs (Fig. 6c and d).

Suggested by Targetscan and PicTar, the online softwares for predicting target gene of microRNA, KLF4 might have a target seed sequence of miR-145. Then we used the luciferase reporter assay to determine whether miR-145 directly target KLF4 in LX-2 cells and found that miR-145 reduced 50–60% luciferase activities of KLF4 3′-UTR expression plasmid, indicating a direct binding of miR-145 (Fig. 6e).

## Discussion

In the present study, the effect of miR-145 on HSC activation and underlying mechanism were investigated. We made a couple of findings: (1) miR-145 were significantly increased in the process of cultured HSCs spontaneously and TGF-β induced activation; (2) miR-145 promoted HSC activation; (3) KLF4 was down-regulated in activated HSCs and in cirrhotic liver tissues; and (4), suppression of KLF4 could be an important molecular basis for miR-145 mediated HSC activation. Collectively, our observation indicated that, via targeting KLF4, miR-145 could be an essential regulator in HSCs activation and in liver cirrhosis.

Recently, miRNAs have emerged as key regulators in chronic liver diseases, including hepatic fibrosis^24^. Differential miRNA expression profiles have been shown in fibrotic liver tissues or HSCs, including rodent injury models (e.g., BDL and CCl4 models, culture-activated HSCs)^3^,^25^,^26^. MiR-19, -29b, were significantly down-regulated in activated HSCs, whereas miR-21, miR-138, and -140 family members were significantly up-regulated^11^,^26^,^27^,^28^. MiR-143/145 was also increased in lung myofibroblasts and stromal fibroblasts of scirrhous type gastric cancer^29^,^30^. Although, recently Zhou *et al*. reported that miR-145 was decreased in activated HSCs^31^. In our study, we found that miR-145 was up-regulated (Fig. 1) both by micro-array and qRT-PCR. We showed that miR-145 was elevated both in culture activated HSCs or TGF-β stimulated activation (Fig. 1). More importantly, miR-145 levels were also significantly increased in CCl_4_ induced cirrhotic liver tissues of rats and natural cirrhotic liver specimen of human patients (Fig. 2). The previous data has been repeated for 3 times independently by 2 persons and has been presented 2 years ago in UEG week. Zhou’s study mainly used T6 and LX2 cell lines while we mainly use the primary rat HSCs. This difference between our study and Zhou’s results could be caused by the difference between cells.

MiRNAs are involved in HSCs activation by modulating the expression of fibrotic markers such as collagens and α-SMA. It has reported that miR-19b decreased COL-I expression^32^, while miR-146a induced α-SMA expression^33^. Our results showed that inhibition of miR-145 repressed COL-I and α-SMA expression, while miR-145 mimics upregulated COL-I and α-SMA expression in HSCs. A miRNA could have multiple target mRNAs, and each mRNA could be targeted by various miRNAs. KLF4 has been reported as one of the main targets of miR-145 in vascular smooth muscle cells^17^. Consistent with these reports, our data has shown an inhibitory effect of miR-145 on KLF4 expression. We also found that KLF4 was the direct target of miR-145 in HSCs. This negative regulation of KLF4 expression could be one of the mechanisms of miR-145 mediated HSC activation.

The function of KLF4 in fibrosis was controversial. As a member of the zinc-finger class of DNA-binding transcription factors, KLF4 was first identified in the epithelial lining of the gut and skin. KLF4 regulates α-SMA transcriptional regulation with discrepant role in different cell types^17^. Zhang *et al*. reported that up-regulation of KLF4 by angiotensin II promoted α-SMA and COL-I expression in cardiac myofibroblasts, while silence of KLF4 by siRNA gave rise to similar results in vascular smooth muscle cells^16^. Marrone *et al*. recently demonstrated that induction of KLF2 in response to simvastatin maintains HSCs in a quiescence status14. While KLF2 siRNA failed to reverse the effect of simvastatin on HSCs activation suggesting other mechanisms may contribute to improve HSCs phenotype. Furthermore, there was a compensatory increment of KLF4 and KLF6. KLF6 was identified to stimulate the activation of HSCs^34^ while the role of KLF4 in HSCs activation is unknown. In the present study, for the first time, we found that KLF4 was down-regulated in both spontaneously and TGF-β induced HSC activation (Fig. 4). More importantly, KLF4 was significantly decreased in cirrhotic liver of patients (Fig. 4e,f). By using CCl_4_ induced rat fibrotic model, we also showed that KLF4 expression was dramatically down-regulated in the cirrhotic liver (Fig. 4g). KLF4 knockdown induced α-SMA and COL-I expression, while overexpressing KLF4 suppressed α-SMA and COL-I expression in HSCs (Fig. 5), demonstrated that KLF4 is a key inhibitory factor for liver fibrosis.

In conclusion, our study demonstrated that miR-145 promoted HSC activation. Repressing KLF4 gene expression could be the possible mechanism, and KLF4 acted as a negative regulator of HSC activation.

## Methods

### Patients and liver tissues collection

From the database of department of Biorepository of the West China Hospital Sichuan University, 16 hepatic cirrhotic liver tissues were selected and 16 liver tissues without liver fibrosis (the remote tissues of liver hemangioma) were enrolled into this study. All the liver tissues stored in department of Biorepository were obtained from patients who underwent liver surgery for a variety of chronic liver diseases in West China Hospital of Sichuan University. The liver samples were stored in liquid nitrogen until use.

All above studies were approved by the Medical Ethics Committee of the West China Hospital of Sichuan University in all of the above-mentioned facilities. All methods were performed in accordance with the related guidelines and regulations. Written informed consent was obtained from all patients at their enrollment.

### Induction of liver cirrhosis by CCl4 and Primary HSCs isolation and culture

Male Sprague-Dawley rats 260–280 g (Animal Center of Sichuan University, China) were used. Two groups of 6 rats each received subcutaneous injection by either 60% CCl_4_ (0.3 ml/kg, 2 times/week) or by vehicle (olive oil) for 8 weeks. Each injection of CCl4 or olive oil was normalized to the current body weight of rat. Rat primary HSCs were isolated from livers of normal male Sprague-Dawley rats (Animal Center of Sichuan University, China) with an optimal body weight of 400–550 g, by *in situ* perfusion of pronase and collagenase and by single-step Nycodenz gradient centrifugation as our previous report^35^.

All animal studies were approved by the Medical Ethics Committee of the West China Hospital of Sichuan University in all of the above-mentioned facilities. All methods were performed in accordance with the guidelines and regulations of Medical Ethics Committee of West China Hospital of Sichuan University.

### Pharmaceutical treatments to HSCs

Rat primary HSCs were grown in DMEM medium (low glucose) with 20% FBS (Hycolon, USA). Passaged rat primary HSCs (3–5 passages) were incubated in DMEM without FBS for 8 hours before treated with 10 ng/mL TGF-β (Peprotech, USA).

The human HSCs cell line LX-2 (gift from Dr. Friedman) were grown in DMEM medium (high glucose) with 10% FBS (Hycolon).

### RNA interference and transfection

Plasmids for expression of KLF4-specific shRNA (pKLF4-shRNAs) and scrambled shRNA were constructed as our previous report. Briefly, three pairs of shRNAs targeting rat KLF4 were designed according to NCBI Gene Bank, and then constructed into the pSuper plasmid by DNA recombination technology. KLF4-expressing plasmid (pKLF4) was obtained from Addgene (the addgene ID is 34593). Culture activated rat primary HSCs within 3–5 passage were transfected with either pKLF4-shRNA or pKLF4 by Lipofectamine^TM^ 2000 (Invitrogen, USA) for 48 hours, according to the manufacturer’s protocol respectively^36^.

The miR-145 mimics and inhibitor were designed and purchased from GeneCopoeia (USA) and transfected into activated HSCs at passages 3–5 at final concentration of 50 μM by Lipofectamine^TM^ 2000.

### Real-time RT-PCR

The levels of mRNA or miRNA were identified by quantitative RT-PCR (qRT-PCR) as reported earlier. Total RNA was extracted from cells or liver tissues from patients and rat with Trizol (Invitrogen, USA), and cDNA was synthesized using a reverse transcription kit (Toyobo, Japan for mRNA and GeneCopoeia, USA for miRNA). qRT-PCR was carried out on CFX96™Real-Time PCR Detection System (BIO-RAD, USA) with fluorescence dye SYBGreen (Toyobo, Japan for mRNA and GeneCopoeia, USA for miRNA). The primer sequence was listed in Table 1. Normalization of gene expression level was conducted with the mRNA level of β-actin. Relative expression values were obtained by normalizing CT values using △△CT method.

### Western blot analysis

Proteins extracted from culture cells, human or rat liver tissue lysates were quantified by VARIOSKAN (Thermo, USA) using a BCA protein assay kit (Thermo/Pierce, USA). Equivalent amounts (20–30 μg) of total protein were separated by 10% SDS-PAGE and transferred to a 0.45 μm PVDF membrane (Roche). The membrane was subsequently blocked with 10% skim milk in TBST solution for 2 h and incubated with primary antibodies at 4 °C overnight. Horseradish peroxidase conjugated goat anti-mouse or anti-rat IgG was used as secondary antibody. The antigen-antibody complexes were detected by Pierce ECL substrate kit (Thermo/Pierce, USA). Specific bands were scanned and quantified by Quantity One software (BioRad, USA). β-actin was used as the loading control.

### Immunohistochemical staining

Liver specimen from 16 cirrhotic patients and 16 non-fibrosis controls were embedded in paraffin and applied for immunohistochemical stain. De-paraffinized antigens were retrieved by heat exposure using a PH8 EDTA-Retriever retrieval steamer for 45 minutes. The endogenous peroxidase was blocked with 3% H_2_O_2_ in methanol for 15 min. The slides were incubated with rabbit anti-human KLF4 (Abcam, HK) at 1:100 dilution for 1 h, incubated with an anti-rabbit HRP-conjugated antibody for 45 min at 37 °C. Then the secondary antibody was added (ChemMateTMEnVision+/HRP) and incubated for 45 min, followed by addition of 50 to 100 μL of diaminobenzidine (DAB). Nuclei were stained with hematoxylin. Hematoxylin and eosin and Sirius Red staining was performed according to standard protocols on paraffin sections.

### Luciferase reporter assay

3′-UTR containing putative miR-145 binding sites of the KLF4 gene was obtained by PCR from LX-2 and was constructed into the pmirGLO Vector (Promega, USA). LX-2 cells were transfected with 50 nM miR-145 mimics or negative control (NC) along with empty pmirGLO vector or KLF4-UTR-pmirGLO by Lipofectamine^TM^ 2000 for 24 hours. Firefly luciferase activity was analyzed by Dua-Glo Luciferase Assay System (Promega) and was normalized to the Renilla luciferase activity.

### Statistical analysis

Data were presented as Means ± SD. Multiple comparisons for different groups were carried out using unpaired t-test or one-way analysis of variance (*ANOVA*) followed by S.N.K. test as post-hoc analysis with SPSS 18.0 software, and *p* < 0.05 was considered statistically significant^37^.

## Additional Information

**How to cite this article**: Men, R. *et al*. MircoRNA-145 promotes activation of hepatic stellate cells via targeting krüppel-like factor 4. *Sci. Rep.* **7**, 40468; doi: 10.1038/srep40468 (2017).

**Publisher's note:** Springer Nature remains neutral with regard to jurisdictional claims in published maps and institutional affiliations.

## Figures and Tables

**Table 1:** List of primer sequences for qRT-PCR.

Gene	Primer Sequences	Product Length (bp)
α-SMA	CCG AGA TCT CAC CGA CTA CC	120
	TCC AGA GCG ACA TAG CAC AG	
Collagen I	ACG TCC TGG TGA AGT TGG TC	118
	TCC AGC AAT ACC CTG AGG TC	
β-actin	ACT ATC GGC AAT GAG CGG TTC	77
	ATG CCA CAG GAT TCC ATA CCC	
KLF4	CAT TCC AAT ACC AAA TCC GAC T	180
	GAG CAT ACA AGG TGG TCT TTC C	
