# Author Correction: MircoRNA-145 promotes activation of hepatic stellate cells via targeting krüppel-like factor 4

**DOI:** 10.1038/s41598-023-36674-4

**Published:** 2023-06-13

**Authors:** Ruoting Men, Maoyao Wen, Mingyue Zhao, Xuelian Dan, Zongze Yang, Wenchao Wu, Maggie Haitian Wang, Xiaojing Liu, Li Yang

**Affiliations:** 1 Division of Gastroenterology & Hepatology, West China Hospital, Sichuan University, Chengdu, 610041 China; 2 Department of Biostatistics, JC School of Public Health and Primary Care, Faculty of Medicine, The Chinese University of Hong Kong, Ma Liu Shui, China; 3 Laboratory of Cardiovascular Diseases, Regenerative Medicine Research Center, West China Hospital, Sichuan University, Chengdu, 610041 China; 4 Creation and Management of a Tumour Bank, West China Hospital of Sichuan University, Chengdu, 610041 Sichuan China

Correction to: *Scientific Reports* 10.1038/srep40468, published online 16 January 2017

This Article contains errors.

As a result of an error during figure assembly, the KLF4 panel in Figure 4b is duplicated in Figure 4e. The corrected Figure 4, with the correct KLF4 and corresponding controls, appears below.

**Figure 4:**
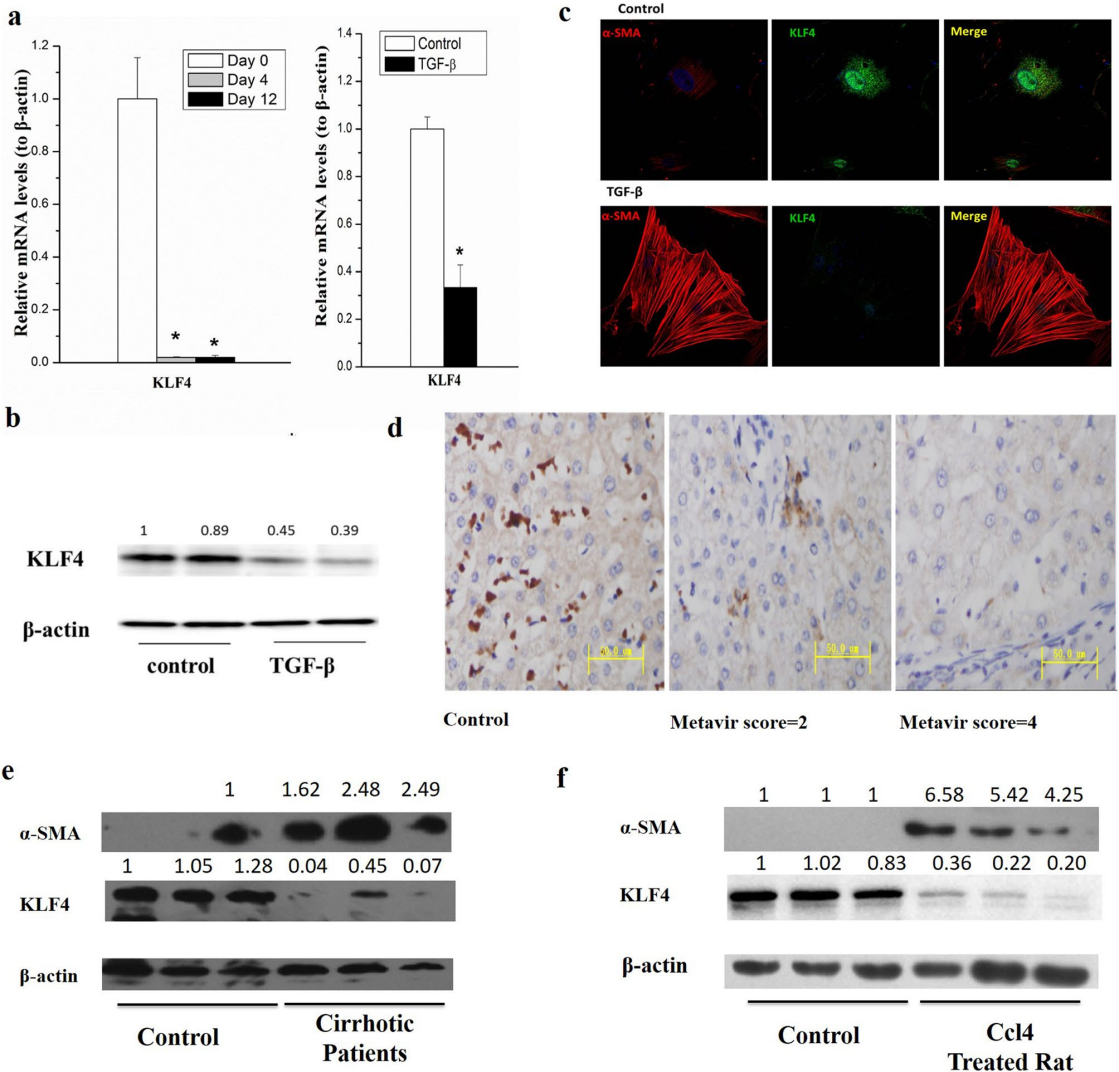
(**a**) The mRNA level of KLF4 dramatically decreased in the process of spontaneous HSC activation (left panel) and primary HSCs treated with 10 ng/mL of TGF-βfor 24 h (right panel). (**b**) KLF4 protein level was significantly decreased in HSCs by treating primary HSC with 10 ng/mL TGF-β for 24 h. (**c**) TGF-β down-regulated KLF4 while upregulatedα-SMA in rat primary HSCs. KLF4 and α-SMA were detected by immunocytochemical staining and pictures were taken with a confocal microscopy. Rat primary HSCs were treated with 10 ng/mL TGF-β for 4 h. (**d**) KLF4 was significantly suppressed in cirrhotic liver of patients as compared with the healthy controls (scored by immunohistochemistry studies, n = 16). (**e**) KLF4 was significantly down-regulated, whileα-SMA was obviously up-regulated in human cirrhotic liver tissues compared with the healthy controls. (**f**) KLF4 was elevated in CCl_4_ induced cirrhotic liver of rats. All experiments were repeated thrice with triplicate samples in each experiment. The relative value of target mRNA/protein to β-actin was set as 1 in the control. Data are presented by mean ± standard deviation. *p < 0.05; **p < 0.01.

